# Accounting for one-channel depletion improves missing value imputation in 2-dye microarray data

**DOI:** 10.1186/1471-2164-9-25

**Published:** 2008-01-19

**Authors:** Cecilia Ritz, Patrik Edén

**Affiliations:** 1 Computational Biology and Biological Physics, Department of Theoretical Physics, Lund University, Sweden

## Abstract

**Background:**

For 2-dye microarray platforms, some missing values may arise from an un-measurably low RNA expression in one channel only. Information of such "one-channel depletion" is so far not included in algorithms for imputation of missing values.

**Results:**

Calculating the mean deviation between imputed values and duplicate controls in five datasets, we show that KNN-based imputation gives a systematic bias of the imputed expression values of one-channel depleted spots. Evaluating the correction of this bias by cross-validation showed that the mean square deviation between imputed values and duplicates were reduced up to 51%, depending on dataset.

**Conclusion:**

By including more information in the imputation step, we more accurately estimate missing expression values.

## Background

Gene expression profiling using microarrays plays an important role in many areas of biology. Microarray data however often contains many missing values. Among the most commonly used computer analysis tools that require imputation of missing values are data dimensionality reducing algorithms such as principal component analysis (PCA) and singular value decomposition, and machine learning algorithms such as support vector machines. Advanced imputation methods have therefore been developed, such as KNNimpute [1], Bayesian PCA [2] and LLSimpute [3], which all are based on correlations between available measurements in the data matrix (samples × reporters). In KNNimpute, *e.g.*, a weighted average of the *K *most similar genes (defined by Euclidean distance) is used to derive an estimate of a missing value in the gene of interest [1]. Missing values and the choice of imputation method has also been shown to affect the significance analysis of differentially expressed genes [4,5].

Missing values can occur due to dust or scratches on the slide, spotting problems or hybridization problems. Obviously problematic spots are manually flagged as missing and are removed from further analysis. It is customary to subtract background intensities from the spot intensity, and this also produces missing values. Negative background-corrected intensities arise if the spot intensity is comparable to the background intensity, either due to contamination of the spot, leakage from neighbouring spots, or from low abundance of dyed cDNA in the reference or sample.

Obviously, the choice of spot quality assessment and transform of intensities of the resulting values will influence the final result of imputation as well as the analysis as a whole. Error models, and transforms other than the logarithm, have been developed, designed to give reliable variance estimates in the absence of replicates [6,7] or generate a well-defined distribution of expression values [8].

However, more heuristic approaches remain in use. One reason could be that many transforms or weights require careful tuning of parameters, which tend to be platform dependent [7], and perhaps even dataset specific. Results from experiments designed to determine such parameters are not always available. Another reason is that the biological variation within traits tends to dominate over technical variation. When the study is large enough to get a reliable sample estimate of the total variation within traits, there is less need for information on technical variation alone. Furthermore, instead of relying on a specific distribution of expression values, *p*-values and false discovery rates [9] are often obtained by non-parametric tests [10] or empirical methods such as permutation tests [11,12].

In heuristic analyses, some spot quality control is still performed, often in terms of threshold values in observables such as spot size, intensity, background variation, or combinations thereof, which are used to flag spots as missing. An undesired feature of this approach is the sharp threshold effects. A spot with an observable, say reference intensity, just below the chosen threshold will be deemed "completely unreliable", while a spot with essentially the same intensity, but just above the threshold will be considered "perfectly reliable".

Different smoothings of threshold effects have therefore been developed [7,13-15]. Smoothing introduces continuous weights, ranging from 0 for missing or "completely unreliable" measurements, to 1 for "perfectly reliable" ones, but also taking on values in between. The chosen weight is related to some commonly used threshold observables and threshold values, typically tuned to be about 1/2 at the otherwise adopted threshold.

Weights *w *associated to the expression values *x *(with *w *= 0 for missing values) can be used to improve imputation [16]. For every spot, measured as well as missing, an imputed value *x*_imp _is calculated and an adjusted value

*x' *= *wx *+ (1 - *w*)*x*_imp_

is used in the subsequent analysis. Thus, missing and "completely unreliable" values *x *are replaced by *x*_imp_, "perfectly reliable" measurements *x *are kept, and spots with weights between 0 and 1 will end up with an expression value somewhere between the imputed and the directly measured value.

Weighted imputation requires a weight definition which ranges from 0 to 1. This property is used in eq. 1 and in the selection of the number of nearest neighbours [16]. The range constraint on the weights excludes weighting schemes that combine an error model estimate of the variance *σ*^2 ^with a weight motivated by maximum likelihood, *w *= 1/*σ*^2^. Instead, weights representing a smoothing of an otherwise adopted threshold filter satisfy the range constraint.

For 2-dye cDNA microarrays, it is common to impute missing values using the data matrix of log intensity ratios. In this approach, no information as to why a measurement is missing is included. It is also possible to impute intensities for each channel separately and form the log ratio from them [5]. Different forms of missing values are then handled differently, but the imputation of a missing intensity is performed without use of the information provided by the other channel of the same spot.

We divided missing values of 2-dye cDNA data into three categories; those that are missing due to a missing sample intensity only (sample depleted spots), a missing reference intensity only (reference depleted spots), or other reasons. We examined if this categorization can be used to improve imputation of expression values. We wanted to investigate imputation of one-channel depleted spots using the best imputation scheme available. We therefore worked with the weighted version presented in [16] and described in our methods section.

## Results and Discussion

### Imputation of one-channel depleted spots is biased

The weighted imputation final result *x' *can be written *x' *= *x*_imp _+ *w*(*x *- *x*_imp_). For one-channel depleted spots, the fact that the opposite channel was measurable provides information which is neglected when we set the weight to 0. For example, sample depleted (sd) spots are those for which the sample channel is missing, while a measurable reference channel indicates a good quality spot, which suggests a low expression. If this qualitative statement "low" is not completely unreliable, warranting a non-zero weight, our final result would be *x' *= *x*_imp _+ "some *w *> 0" × ("low" - *x*_imp_).

To quantify this pseudo-mathematical formula, we asked ourselves if "low" meant "lower than *x*_imp_", at least on average, for sd spots. If so, the relation can be written *x' *= *x*_imp _+ "some negative correction". We used datasets with duplicate measurements to test this. For some of the duplicates, one measurement is sd while the other is known. We used the known duplicate as the best available estimate of the value that the sd spot should have, and compared this control value with the imputed value.

Similarly, reference depleted (rd) spots are those for which the sample channel is measurable, in spite of the poor spot quality indicated by a missing reference, suggesting a high expression. We used the same datasets and compared the imputed values of rd spots with known duplicate controls, to test if the qualitative statements "some *w *> 0" and "high" motivated a positive correction of the imputed value for rd spots. Table 1 shows that for one-channel depleted spots, the imputed value has a mean deviation (md) from the duplicate much larger than expected by random, while the bias for other spots is more comparable to random expectation. Furthermore, the md signs are in agreement with the hypotheses that sample- and reference depletion provide useful information about down- and up-regulation, respectively.

**Table 1:** Mean deviation and mean squared deviation. For five datasets and three categories of missing values, imputed values using WeNNI are compared to duplicate controls. The first columns show the number of spots (*N*), the uncorrected mean squared deviation (msd, defined in eq. 3) and the uncorrected mean deviation (md, defined in eq. 4) relative to random expectation, $\sqrt{\text{msd}/N}$. The sd/rd spots are systematically over/under-estimated, with a deviation much larger than random expectation. For the other missing spots, the deviation varies in sign and is more comparable to random expectation. For sd and rd spots, validation results of a constant correction are also shown, revealing mean deviations well within random expectation, except for the MEC tumours dataset (but there it is noticeably lower than the uncorrected result). The msd is reduced compared to the uncorrected result. The relative reduction is reported in the rightmost column.

		Un-corrected		Corrected (validation result)		
Dataset	*N*	msd	$\frac{\text{md}}{\sqrt{\text{msd}/N}}$	$\frac{\text{md}}{\sqrt{\text{msd}/N}}$	msd	msd decrease
breast cancer						
sd	11733	0.74	30	0.02	0.69	6.1%
rd	48523	1.02	-100	0.3	0.81	21%
other	6865	0.64	-8			
lymphoma						
sd	1707	1.52	30	1	0.74	51%
rd	6541	1.23	-50	-0.3	0.73	40%
other	41499	0.54	-10			
MEC cell lines						
sd	4284	0.98	20	-0.3	0.86	12%
rd	942	1.33	-10	0.2	1.14	14%
other	15584	0.27	-1			
MEC tumours						
sd	12899	1.24	50	-8	1.00	19%
rd	3376	1.87	-20	-1	1.60	15%
other	21616	0.52	8			
melanoma						
sd	10498	1.92	10	0.2	1.89	1.6%
rd	68503	1.99	-100	0.1	1.72	14%
other	4754	2.09	-4			

Thus, though we could not *a priori *be certain that the pseudo-mathematical expression "some *w *> 0" × ("low" - *x*_imp_) implies "some negative correction", we found that it does so, in five different datasets produced on different platforms by different experimental groups, investigating both primary tissue and cell lines.

Above, we referred to the weighted scheme to motivate why we looked for an imputation bias, but it should be pointed out that the result, that bias exists, is more general. We also performed imputation with binarized weights as in [16], so that all weights below 0.5 were set to 0 and all others to 1. This corresponds to unweighted KNNimpute, and we then found similar biases (data not shown).

### Corrections calibrated on training set duplicate controls improves imputation in validation set

The next obvious step was to design and evaluate a correction of the observed bias. In datasets with duplicates, the simplest possible correction is just a constant shift removing the bias found in duplicates where one measurement is sd (rd) while the other is known. The calibrated correction can then be applied to all sd(rd) spots.

Duplicates used to calibrate the correction are useless as controls. Therefore, we tested the constant shift correction in a cross-validation scheme described in methods. The md in the validation set was found to be comparable to random expectation (see Table 1).

A change of md to a new md' will change the mean square deviation (msd) by (md*'*)^2 ^- (md)^2^. Table 1 shows that this corresponds to a reduction of msd varying from 1.6% to 51%, depending on dataset, with a median of 14%. Thus, a constant shift removing the mean deviation (md) on a subset of samples (the training set) reduces the mean square deviation (msd) on another subset of samples (the validation set). In summary, a correction calibrated on a subset of samples (the training set) can be used to essentially remove the bias and reduce the msd in another subset of samples (the validation set).

### Corrected imputation increases overall variance without reducing signals for de-regulation

Next, we wanted to assess the impact of bias correction for finding differentially expressed reporters. We therefore tested imputation with and without missing value categorization in a more realistic analysis, where duplicates were being merged rather than kept apart for imputation control. We combined merging and imputation as described in methods.

**Figure 1:**
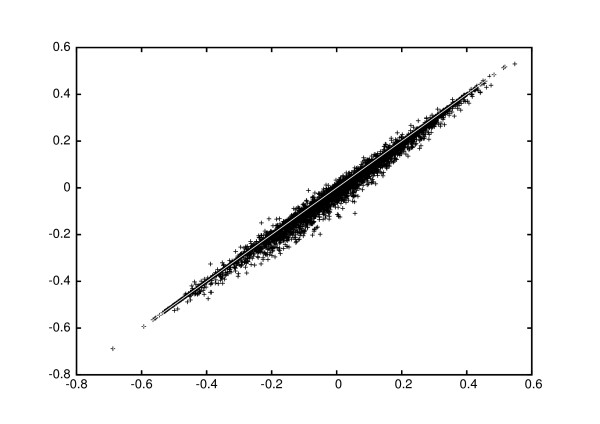
**Pearson scores after different imputation methods**. Pearson scores for clinical outcome in the lymphoma dataset. The Pearson scores calculated with and without correction is shown on the *x*- and *y*-axis, respectively. The diagonal line is inserted to guide the eye. There is no significant change in the distribution of score magnitudes.

**Figure 2:**
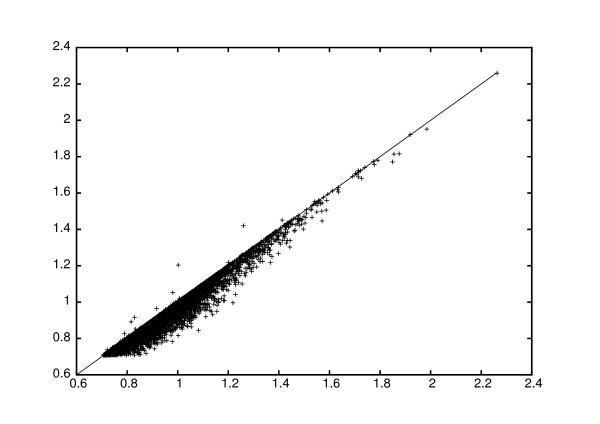
**Reporter standard deviation after different imputation methods**. Reporter standard deviation in the lymphoma dataset. The standard deviation of expression, calculated with and without correction, is shown on the *x*- and *y*-axis, respectively. The diagonal line is inserted to guide the eye. The corrected imputation method results in larger variations of expression.

For the lymphoma dataset, we constructed reporter lists for the 5000 reporters with largest variation across arrays after WeNNI, with and without the modification. The reporters were ranked on Pearson correlation to clinical outcome. In this dataset, the number of sd and rd spots correlated with good and bad clinical outcome, respectively, with an odds ratio of 13.3. This correlation manifested itself with a shift towards more positive Pearson scores with modified imputation, see Figure 1. However, the distribution of score magnitudes did not change much, and the false discovery rates [9], estimated from random sample label permutations, gave also very similar results for the two approaches (data not shown). We also studied how the reporter standard deviation changed when applying the correction, and found that the variation increased for the vast majority of the modified reporters, see Figure 2. This implies that more reporters would survive a variation filter after correction, and would be kept for further analysis, thus allowing for a better sensitivity. The same analysis was also performed in the MEC tumour dataset, with very similar results.

## Conclusion

We show that conventional imputation of 2-dye CDNA data gives biased estimates of expression for missing values where only one channel is missing. We present the simplest possible correction, just a constant shift calibrated on duplicate controls, which can be used to improve imputation for these one-channel depleted spots.

The method does not apply to experiments where the two dyes are used for pairwise comparison of biological traits, but it is applicable to all 2-dye cDNA microarray data with a common reference on all arrays, and with at least some duplicates, to allow for calibration.

In principle, common reference experiments with dye swap arrays can be corrected for bias in one-channel depleted spots, as long as the reference channel is properly identified on each array. With the datasets available in this study, a possible benefit from dye-specific corrections could not be investigated.

We speculate that the missing value categorization is more useful in smaller datasets where the KNN-based method is less likely to find the correlations needed for a successful imputation. This is supported by Table 1, where the three smallest datasets lymphoma, MEC cell lines and MEC tumours show the greatest improvement. Furthermore, datasets with homogeneous classes, such as extensively FACS-sorted cell populations, or replicates of transfected cell-lines, are probably more likely to contain those consistently strong down-regulated genes that will be correctly characterized as sample depleted. In fact, we can see that in the MEC tumour and cell line datasets where the samples represent distinct gene expression phenotypes, the sd spots outnumber the rd spots in contrast to the other datasets, where rd spots are more common than sd spots.

In these cases, the imputation method proposed here may not only help create complete data matrices for algorithmic purposes, but may also identify strongly down-regulated genes that would not be found to be differentially expressed by conventional imputation.

## Methods

### Data

We used five datasets to examine imputation of one-channel depleted spots. Two of them have already been used for evaluation of imputation methods [16]: the melanoma and the breast cancer datasets. The melanoma dataset [17] contains 61 samples obtained from cell lines. Spots representing 19,200 unique reporters were printed in duplicate across two slides.

The breast cancer dataset is a subset of a larger ongoing study, and it contains 55 tumour samples. The number of spots on each array was 55,488, and except for a small number of control spots, all reporters were printed in duplicates on the same slide, which results in approximately 27 k unique reporters. The lymphoma data is also a subset of a larger ongoing study [18], investigating extreme clinical outcomes (complete cure or primary failure). The dataset uses the same array platform as the breast cancer dataset. The mycoepidermoid carcinoma (MEC) dataset consists of 11 tumour samples and 6 differently transfected cell-line experiments, which are part of an ongoing study of the disease. Each array contained 36,288 spots and, except for a few control spots, each spot represented a unique reporter. Every tumour and cell-line sample was hybridized on duplicate arrays. The primary tumour and transfected cell-line material address slightly different biological questions and are here treated as two different datasets.

In the melanoma dataset, the reference common to all arrays was derived from one cell line. In all other studies described here, the reference used was the Universal Human Reference RNA (Stratagene, La Jolla, CA).

Measurements where the background subtracted intensity is zero or negative were treated as missing. As we adopted a weighted approach which penalizes low intensities, we needed to treat as missing only the non-positive intensities, for which we could not make the conventional log transform. If the spot was flagged during image analysis as a bad measurement, the intensity values in both channels in that spot was also considered missing. Only in the MEC and lymphoma datasets were there missing values due to flagged spots.

All datasets contained duplicate measurements, either printed on the same array (breast cancer and lymphoma) or on separate arrays (melanoma, MEC). Duplicate measurements were separated into two datasets A and B. The data was filtered before the analysis to contain reporters with at most 50% missing values in any of the duplicates. We then defined one-channel depleted spots to be the spots where the intensity in one of the channels was missing or non-positive after background subtraction.

The differences in experimental setups (cell lines or tumours, duplicates printed on the same slide or different slides, and various sizes) give more reliable evaluation of the method, and might help identify the type of dataset for which the method is most useful.

### Weighted imputation

In weighted imputation, all spots with *w *< 1 are affected by imputation to some degree. Spots flagged as missing still get a final expression equal to the imputed value, *x' *= *x*_imp_, but also for these spots, the result depends on the choice of weight function, since the weights of the full data matrix influence the calculation of *x*_imp _[16].

We chose to work with a weight which represents a smoothing of a simple filter in signal to background noise, SNR, in channels 1 and 2:

$$w=\frac{1}{1+\beta^{2}\left(\frac{1}{{\text{SNE}}_{1}^{2}}+\frac{1}{{\text{SNR}}_{2}^{2}}\right)}.$$

A SNR filter is motivated by the background noise contribution to the variance of the log intensity ratio. If the mean background intensity *I*_*bg *_is modified by an additive random variable *ε*_*bg *_with mean 0 and variance $\sigma_{bg}^{2}$, the first order Taylor expansion of the log background subtracted intensity is log(*I*) → log(*I*_*fg *_- *I*_*bg *_- *ε*_*bg*_) ≈ log(*I*) - *ε*_*bg*_/*I*. This corresponds to an additive random variable with variance $\sigma_{bg}^{2}$/*I*^2^, which is 1/SNR^2^, apart from a constant factor reflecting the difference between natural logarithm and 2-logarithm, and the effective number of independent pixels in the background [7]. The background noise contribution to the log ratio of intensities in channel 1 and 2 is thus proportional to $1/{\text{SNR}}_{1}^{2}+1/{\text{SNR}}_{2}^{2}$.

A SNR filter can be supplemented with filters in other variables, and much more elaborate spot quality tests exist [13,15,19,20], which could be turned into smooth weights confined between 0 and 1 (as was done in [13,15]). However, the simple SNR-based weight is, so far, the only weight that has been thoroughly investigated in imputation context, where it has been shown to improve results considerably [16]. The usefulness of this simple weight is illustrated by the fact that state-of-the-art unweighted imputation schemes, which take correlations in the data matrix into account, are outperformed not only by weighted KNN imputation, but also by a weighted version of the simple row average imputation [16], which in its un-weighted form is substandard.

We set *β *= 0.3, which is shown to give good imputation in [16], where it is also observed that the final result is rather robust against changes of *β *in the range 0.1 – 1.

### Evaluation of imputation

The performance of an imputation method is usually evaluated with the mean squared deviation (msd) between imputed values and controls,

$$\text{msd}=\frac{1}{N-1}\sum_{i}{\left(x_{\text{imp}}-x_{\text{control}}\right)}_{i}^{2},$$

where *i *runs over all *N *imputed spots. The controls can be either artificially removed measurements [1] or duplicates [16]. We worked with duplicates, since artificial removal of well-measured spots prevents missing value categorization.

Bias in the imputation can be detected by the mean deviation (md)

$$\text{md}=\frac{1}{N}\sum_{i}{\left(x_{\text{imp}}-x_{\text{control}}\right)}_{i}.$$

Without bias, the expected magnitude of the md between imputed values and duplicate controls is of the order $\sqrt{\text{msd}/N}$. For three types of missing values (sample depleted, reference depleted and other) we compared md to $\sqrt{\text{msd}/N}$. We used 10 neighbours in the WeNNI algorithm, which has been shown to give good imputation results [16].

Results for both data halves A and B (with the other as control) were averaged, weighted by the number of relevant spots in each half.

### Cross validation of correction

We validated the bias correction using three-fold cross-validation, where the samples were split into three groups and one group was left out one at a time. The bias for sd and rd spots, respectively, was calculated in the remaining two thirds of the samples. We then modified the imputed values of one-channel depleted spots by a constant shift removing this bias. The mean deviation and the mean square deviation was then calculated in the third of the data that had not been used to determine the bias. This was repeated ten times, each time with a new random partition of the three groups, in both data halves A and B (with the other as control). The final result was obtained as the average of all 60 validation results, weighted by the number of relevant spots in each validation set.

### Replicate merging

Separating duplicates into sets *A *and *B *is useful for evaluating imputation methods, but in general, merging of replicates is preferable. Having found the information about sample and reference depletion useful, we wanted to include it in the analysis also after merges.

In addition to a merged expression value, we assigned to the merged result a combination of three flags: "known", "sd" and "rd". The flags were used to remember the type of replicates that where part of the merge. However, if both sd and rd spots were among the replicates, giving conflicting information suggesting both low and high expression, we removed those contradictory flags. This lead to six possible flag constellations after the merge: "known and sd", "known and rd", "known", "sd", "rd", and "none". For the "known and sd" spots, imputed values were calculated and the known values were used to calibrate a constant correction of imputation bias for sd spots. The correction was then applied to the imputed value of all merged spots of the type "sd". A corresponding correction calibrated on "known and rd" reporters was applied to the imputed values of the "rd" reporters.

In principle, missing value categories can be used in this way for all imputation schemes, also un-weighted ones. However, we adopted weighted imputation also after merge, partly because of its strong performance in general [16], but also because it allowed us to include the information about one-channel depletion in the final value of merges of type "known and sd" and "known and rd". Their final value *x' *= *x *+ (1 - *w*)(*x*_imp _- *x*) was modified by the calibrated correction used to adjust *x*_imp_. As desired, this modification of *x*_imp _has negligible effect on *x' *when the known replicate value *x *is of good quality.

In order to use the weighted imputation scheme after a merge, we needed for each set of replicates not only a weighted mean expression

$$x=\frac{\sum_{i}x_{i}w_{i}}{\sum_{i}w_{i}}$$

(where *x*_*i *_and *w*_*i *_are the expression value and weight of replicate *i*) but also a merged quality weight *w *associated with each merged expression *x*.

The weight *w *must be within range 0 to 1 to be applicable in weighted imputation. We satisfy this constraint by using the form *w *= 1/(1 + *f*), where *f *is a non-negative function of replicate weights and expression values. If only one replicate is available, we must have *w *= *w*_1_. This implies that *f *for a single replicate must be (1 - *w*_1_)/*w*_1_.

If replicates agree completely (all *x*_*i *_= *x*), *w *should be larger than the largest weight *w*_max _among the replicates (since confirming measurements improve the reliability of *x*), but if an added replicate has a weight much smaller than *w*_max_, its effect on *w *should be negligible. These two criteria are met by generalizing the *f *for a single replicate to *f *= 1/∑_*i*_[*w*_*i*_/(1 - *w*_*i*_)].

Finally, it is reasonable to suppress the weight *w *if replicate measurements disagree. The weight then represents a smoothing of a conceivable filter in replicate variance, represented by a variance estimate ${\hat{\sigma }}_{x}^{2}$.

We add a term proportional to this variance to get a weight

$$w={\left[1+\frac{1}{\sum_{i}w_{i}/(1-w_{i})}+\beta^{2}{\hat{\sigma }}_{x}^{2}\right]}^{-1}.$$

For merges with at least one high-quality replicate (*w*_*i *_≈ 1) the weight is *w *≈ 1/(1 + *β*^2^${\hat{\sigma }}_{x}^{2}$), which represents a smoothing of a filter flagging all merges with a variance estimate above 1/*β^2^*as missing. To arrive at an expression for the variance estimate ${\hat{\sigma }}_{x}^{2}$, we reasoned as follows. If all replicate values *x*_*i *_are obtained from random distributions with the same mean *μ *but possibly different variances $\sigma_{i}^{2}$, the variance of *x *is $\sigma_{x}^{2}=\sum_{i}w_{i}^{2}\sigma_{i}^{2}/{\left(w_{\text{sum}}\right)}^{2}$, where *w*_sum _= ∑_*i*_*w*_*i*_. The sample estimate $\sum_{i}w_{i}^{2}{\left(x_{i}-\mu \right)}^{2}/{\left(w_{\text{sum}}\right)}^{2}$ has expectation value $\sigma_{x}^{2}$, but is not available when *μ *is unknown. Replacing *μ *by the sample average *x *creates a bias in variance estimation, which in the unweighted case is corrected by a factor *n*/(*n *- 1), where *n *is the number of replicates. This correction does not generalize well to the weighted case, mainly because the generalization *n *= *w*_sum _creates *n *that can be smaller than 1. An alternative generalization of *n *= *w*_sum_/*w*_max _[7] ensures *n *≤ 1, but *n *can be arbitrarily close to 1 when one weight dominates (*w*_sum _→ *w*_max_).

A good sample estimate of the variance should have the correct expectation value $\sigma_{x}^{2}$ when *w*_sum _≫ *w*_max_. For merging purposes it is also essential that it vanishes as *w*_sum _→ *w*_max_. Otherwise a duplicate with vanishing weight has a non-vanishing effect on *w*. We used the estimate

$${\hat{\sigma }}_{x}^{2}=\frac{\sum_{i}w_{i}^{2}{\left(x_{i}-x\right)}^{2}}{{\left(\sum_{i}w_{i}\right)}^{2}},$$

since its expectation value is bounded by

$${\left(1-\frac{w_{\max}}{w_{\text{sum}}}\right)}^{2}\leq \frac{\langle {\hat{\sigma }}_{x}^{2}\rangle }{\sigma_{x}^{2}}\leq 1+\frac{w_{\max}}{w_{\text{sum}}}$$

and the estimate itself by

$${\hat{\sigma }}_{x}^{2}\leq {\left(1-\frac{w_{\max}}{w_{\text{sum}}}\right)}^{2}\sum_{i}{\left(x_{i}-X_{i}\right)}^{2},$$

where *X*_*i *_is the weighted average with replicate *i *omitted, *X*_*i *_= ∑_*j*≠*i*_*w*_*j*_*x*_*j*_/∑_*j*≠*i*_*w*_*j*_. The first range shows that $\langle {\hat{\sigma }}_{x}^{2}\rangle \to \sigma_{x}^{2}$ when *w*_sum _≫ *w*_max_, the second range that ${\hat{\sigma }}_{x}^{2}$ → 0 when *w*_sum _→ *w*_max_.

## Authors' contributions

Both authors contributed to the development of algorithms and evaluation methods. CR performed most of the implementation and result collection.
